# Supplementary material for: Content-rich biological network constructed by mining PubMed abstracts
Source: BMC Bioinformatics. 2004 Oct 8;5:147. doi: 10.1186/1471-2105-5-147 (PMC528731; doi:10.1186/1471-2105-5-147)
Supplement: Additional File 5 — The original Chilibot query results of the term "long-term potentiation (LTP)" and 22 other terms, limiting the latest references analyzed to the years 1990, 1995, 2000, and 2004. [file 1471-2105-5-147-S5.bz2 › chilibotAdditionalFile5/ltp1995/html/CAMKII_CAMKIV.html]

 


 **CAMKII** and **CAMKIV** 
  
Found 2 abstracts in PubMed,  **2 abstracts were retrieved and analyzed**.  


---

 Search Google  |
 PDF files only 
|  EDU domain only 

---

**Interactive relationship** (e.g. stimulation, inhibition, etc)

- The results indicate that  **CaMKIV**  is much more potent than  **CaMKII**  in activating CREB in three different cell lines.  Ref: 7958915 Genes Dev, 1994
- Because both  **CaMKII**  and  **CaMKIV**  can phosphorylate CREB, we pursued further the mechanism by which  **CaMKII**  and  **CaMKIV**  differentially regulate CREB activity.  Ref: 7958915 Genes Dev, 1994

**Parallel relationship** (e.g. studied together, co-existance, homology, etc.)

- Examination of the subcellular distribution of transiently expressed  **CaMKIV**  and  **CaMKII**  reveals that only  **CaMKIV**  enters the nucleus.  Ref: 8065343 Mol Cell Biol, 1994
- The roles of two multifunctional calcium calmodulin dependent protein kinases,  **CaMKIV**  and  **CaMKII** , were examined in transient transfection studies that utilized either the full length or the constitutively active forms of these kinases.  Ref: 7958915 Genes Dev, 1994
- When Ser142 was mutated to alanine, CREB was activated by  **CaMKII** , as well as by  **CaMKIV** .  Ref: 7958915 Genes Dev, 1994
